# *In vitro* Models to Evaluate Drug-Induced Hypersensitivity: Potential Test Based on Activation of Dendritic Cells

**DOI:** 10.3389/fphar.2016.00204

**Published:** 2016-07-12

**Authors:** Valentina Galbiati, Angela Papale, Elena Kummer, Emanuela Corsini

**Affiliations:** Scienze Farmacologiche e Biomolecolari, Università degli Studi di MilanoMilan, Italy

**Keywords:** CD86, ROS, alternative methods, drug hypersensitivity, *in vitro* methods, dendritic cell activation

## Abstract

Hypersensitivity drug reactions (HDRs) are the adverse effect of pharmaceuticals that clinically resemble allergy. HDRs account for approximately 1/6 of drug-induced adverse effects, and include immune-mediated (“allergic”) and non-immune-mediated (“pseudo allergic”) reactions. In recent years, the severe and unpredicted drug adverse events clearly indicate that the immune system can be a critical target of drugs. Enhanced prediction in preclinical safety evaluation is, therefore, crucial. Nowadays, there are no validated *in vitro* or *in vivo* methods to screen the sensitizing potential of drugs in the pre-clinical phase. The problem of non-predictability of immunologically-based hypersensitivity reactions is related to the lack of appropriate experimental models rather than to the lack of -understanding of the adverse phenomenon. We recently established experimental conditions and markers to correctly identify drug associated with *in vivo* hypersensitivity reactions using THP-1 cells and IL-8 production, CD86 and CD54 expression. The proposed *in vitro* method benefits from a rationalistic approach with the idea that allergenic drugs share with chemical allergens common mechanisms of cell activation. This assay can be easily incorporated into drug development for hazard identification of drugs, which may have the potential to cause *in vivo* hypersensitivity reactions. The purpose of this review is to assess the state of the art of *in vitro* models to assess the allergenic potential of drugs based on the activation of dendritic cells.

## Introduction

Adverse drug reactions (ADRs) are defined by the World Health Organization as “any noxious, unintended, and undesired effect of a drug that occurs at doses used for prevention, diagnosis, or treatment”. ADRs can be categorized into type A—predictable (about 80% of all ADRs), and type B—unpredictable, reactions. Type A (predictable) reactions are usually dose-dependent, related to the known pharmacologic actions of the drug, and occur in otherwise health subject while type B (unpredictable) reactions are generally dose independent, are unrelated to the pharmacologic actions of the drugs, and occur only in susceptible subjects. Unpredictable reactions are subdivided into drug intolerance, drug idiosyncrasy, drug allergy and pseudo-allergic reactions (Khan and Solensky, 2010). It is difficult to distinguish between pseudo allergic reactions and true immunologically mediated allergic reactions, but the first one lack immunological specificity (Warrington and Silviu-Dan, 2011). The Gell and Coomb's classified hypersensitivity reactions into 4 types and this classification system includes:

- Type I reactions: immediate-type reactions mediate by immunoglobulin E (IgE) antibodies. Drug IgE complex bind to mast cells with release of histamine and inflammatory mediators, resulting in anaphylaxis, urticarial, angioedema, bronchospasm. Examples of drugs include penicillin and cephalosporins.
- Type II reactions: cytotoxic reactions mediated by drug-specific immunoglobulin G (IgG) or immunoglobulin M (IgM) antibodies. Specific IgG or IgM antibodies are directed at drug-hapten coated cells, resulting in anemia, cytopenia, thrombocytopenia. Examples of drugs includes hydralazine, methyldopa and procainamide.
- Type III: immune-complex reactions. Tissue deposition of drug-antibody complexes result in complement activation and inflammation (i.e., serum sickness, vasculitis, fever, rash, arthralgia, lupus). Examples of drugs include penicillin, sulphonamides, hydralazine and procainamide.
- Type IV reactions: delayed-type hypersensitivity reactions mediated by cellular immune mechanisms. MHC presentation of drugs to T cells results in the release of cytokine and inflammatory mediators, which recruit inflammatory cells (i.e., contact sensitivity, skin rashes, organ-tissue damage). Type IV reactions can be divided in subcategories, with the activation and recruitment of monocytes, eosinophils, CD4+ or CD8+ T cells, and neutrophils (Pichler, 2003a; Riedl and Casillas, 2003; Warrington and Silviu-Dan, 2011). Examples of drugs include neomycin, penicillin and benzocaine.

*In vivo* tests like patch, prick and intra-cutaneous tests often do not yield positive reactions for the diagnosis of drug allergy and lacks optimal sensitivity that still remains a major problem in daily clinical practice (Sachs et al., 2002). For these reasons, *in vitro* stimulation could be required as a complementary diagnostic test, as emerged from some studies (Torres et al., 2003; Romano et al., 2004; Sachs et al., 2004).

Nowadays there are no validated *in vivo* or *in vitro* methods for assessing the sensitizing potential of a drug during the pre-clinical phase, although the important adverse reactions directly linked to immune-mediated hypersensitivity and autoimmunity reactions. Available *in vitro* tests mainly refer to the effector phase of immediate-type drug allergic reactions, such as the CAST-ELISA® (Kubota et al., 1997), which is based on the presence of specific IgE antibodies and the BASO-Test® (Pâris-Köhler et al., 2000), based on the activation of basophils like.

Currently, the popliteal lymph node assay (PLNA), or its modifications, can be used in research studies for the identification of drugs, which may be potential allergens, or may cause autoimmunogenic reactions (Warbrick et al., 2001). However, no reliable models or general strategy and assays (including the PLNA) are at present available (or validated) and requested by regulatory agencies. PLNA appears to be very useful for the assessment of the potential of drug to initiate an immune response. The simplest, the primary PLNA, measures popliteal lymph node hyperplasia after subcutaneous injection of a chemical into the footpad of a mouse or rat. The PLN-index is obtained with the ratio of weight or cell number of the draining lymph node of the chemical-treated animals over the vehicle-treated animals (Pieters, 2001). With the primary PLNA, the involvement of specific T cells cannot be assessed; therefore, in a previously sensitized animal, a secondary PLNA must be performed by measuring the PLN index of a chemical. In this assay, purified and irradiated T cells from sensitized syngeneic donors are injected subcutaneously into the footpad of naïve acceptor mice 1 day before injection of the chemical or its active metabolite. Finally, the modified PLNA, defined reporter antigens TNP-OVA (T cell-dependent antigen) and TNP-Ficoll (T cell-independent antigen) are used to distinguish between sensitizing and non-sensitizing (IgG1-response or not to TNP-Ficoll, respectively) drugs (Albers et al., 1997). The primary PLNA is particularly suitable as preclinical screening assay, but it cannot distinguish between strong irritants and sensitizing compounds. Also the more complicated modified PLNA may be used as screening assay, and it has additional advantages over the primary PLNA including (a) the parameters measured (antibody production) are immunologically more relevant than lymph node weight or cell number; (b) the immune response can be measured without knowing the nature of the neo-antigens and it can discriminate between sensitizing, non-sensitizing and complete innocent chemicals. In any case, some of the compounds known to cause immune adverse effects in humans, however, failed to induce a positive PLNA response, leading to refinements of the technique to include pretreatment with enzyme inducers, depletion of CD4+ T cells or additional endpoints such as histological examination, lymphocyte subset analysis and cytokine fingerprinting (Ravel and Descotes, 2005).

It is well known that immunological adverse drug reactions are rare (Gruchalla, 2001; Pichler, 2003b) but anyway they are generally able to cause a lot of discomfort to patients and may indeed be really dangerous. The withdrawal from the market of drug is also an important economic issue due to the extremely high costs associated with the development of a drug (Pieters, 2007). The development of alternative *in vitro* assays to detect the sensitization potential during the development phase of a drug would increase safety and possibly reduces the risk of market withdrawal (Corti et al., 2015).

Traditional drugs have low molecular weights (<1000 Da) and as a such they are too small to be “seen” by T cells. Therefore, low molecular weight compounds have first to bind to a protein before they will become visible to T cells (Weltzien et al., 1996; Pichler, 2002). In addition, drugs may alter protein structure, the process of antigen presentation, or may, by causing cellular or organ damage, release auto-antigens (e.g., DNA or histones) for which no tolerance exists (Pieters, 2007), favoring the development of hypersensitivity reactions.

This review will focus on the state of the art of available *in vitro* models to assess the potential of drugs to induce hypersensitivity for diagnostic purposes and on the potential *in vitro* test for the pre-clinical assessment.

## State of the art of *in vitro* models to assess drug-inducing hypersensitivity

**Figure 1:**
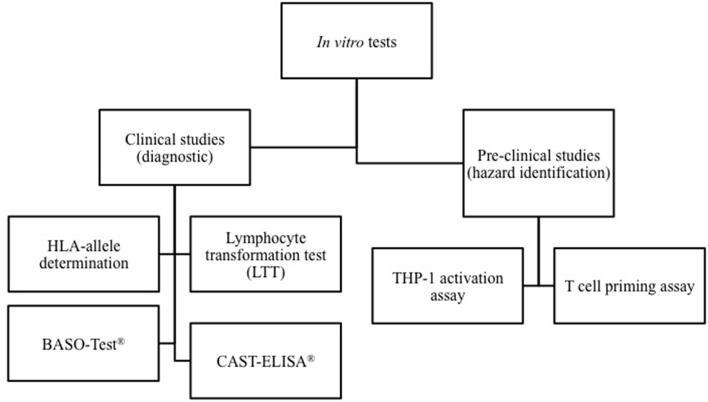
**Classification of *in vitro* models able to assess drug-inducing hypersensitivity reactions**. *In vitro* diagnostic tests can be divided in test able to identify the culprit drugs at the resolution of the reaction, and methods that allow to determining the individual risk of HDR before drug administration. On the other hand, some *in vitro* methods may be used in the pre-clinical phase of drug development for hazard identification of potential to induce hypersensitivity reactions.

The cells involved and mediators released during the different phases of hypersensitivity reactions can be assessed using *in vitro* diagnostic tests. The methods used for the diagnosis of HDR depend on the mechanism involved and the kinetic of the reaction. As shown in Figure 1, *in vitro* diagnostic tests can be divided in test able to identify the drugs but only at the resolution of the hypersensitivity reaction, and in *in vitro* assays, which allow to determining the HDR risks before drug administration.

Is important to mention that *in vitro* tests for the identification of non-immediate reactions (NIR) are not commercially available and therefore standardization is not possible (Mayorga et al., 2016).

## *In vitro* diagnostic tests

### HLA-allele determination

The human leukocyte antigen (HLA) system is a gene complex encoding the major histocompatibility complex (MHC) proteins in humans. HLA genes are highly polymorphic, which means that they have many different alleles, allowing them to fine-tune the adaptive immune system. HLA genotyping is based on reverse sequence-specific oligonucleotide-polymerase chain reaction using DNA from peripheral blood.

Pharmacogenetic testing is not widely used in routine clinical practice to optimize drug choice or clinical management (Philipps, 2006). This gap between scientific knowledge and clinical application may be explained by the fact that the successful incorporation of a pharmacogenetic test into routine practice requires a combination of high-level evidence that can be generalized to diverse clinical setting, wide-spread availability of cost-effective and reliable laboratory tests, and effective strategies incorporate testing into routine clinical practice (Mallal et al., 2008).

Several clinical studies, as reported in the review by Mayorga et al. (2016), correlate hypersensitivity reaction caused by pharmaceuticals with the presence of different HLA allele. In particular HLA-B^*^57:01 has been found to be associated with abacavir hypersensitivity; for carbamazepine, the most important association has been established with HLA-B^*^15:02 and HLA-A31:01, while HLA-B^*^58:01 allele has been associated with allopurinol hypersensitivity.

Prospective HLA-allele screening may be widely useful, but there are some controversial points like the cost-effectiveness of the test, that depend on several estimates that vary among populations, the health care setting, and also the availability of appropriate laboratory assays (Martin et al., 2006; Hammond et al., 2007).

### Lymphocyte transformation test (LTT)

The LTT measures the *in vitro* proliferative response of T cells that emerge from the clonal expansion of naïve T cell, after drug exposure (Nyfeler and Pichler, 1997; Lanzavecchia and Sallusto, 2000). The proliferative response of lymphocytes is measured by the incorporation of ^3^H-thymidine during DNA synthesis or by carboxyfluorescein diacetate succimidyl ester (CFSE). The most widely studied drugs are betalactams (Mayorga et al., 2016). The LTT limits are represented by the use of radioactivity, which limits the application of the method to specific research laboratories, it is a quite long procedure, the sensitivity is quite low (60–70%), and it depends upon the conditions employed (Nyfeler and Pichler, 1997; Romano et al., 1997; Beeler and Pichler, 2007).

### Enzyme-linked immunosorbent spot (ELISpot) assay

ELISpot is a technique used to determine the number of cells able to produce cytokines and cytotoxic markers after their activation by the drug or its metabolites (Sullivan et al., 2015). Clinical studies report the use of IFN-γ ELISpot to diagnose non-immediate reactions to betalactames; granzyme B and granulysin ELISpot for evaluating severe cutaneous reactions induced by amoxicillin, ciprofloxacin, carbamazepine, sulphonamides, allopurinol, mefenamic acid, oxipurinol, and lamotrigine (Zawodniak et al., 2010; Porebski et al., 2013). One advantage of this *in vitro* test is that drug-reactive T cells remain detectable for long time after the reaction and could be appropriate for high throughput screening but to improve the accuracy of the test, two or more cytokines determination could be necessary.

### Cell markers and cytokine release

After drug stimulation, T-cells express or up-regulate a number of surface molecules and produce different inflammatory mediators. Cytokine expression and secretion can be evaluated by several methods such as reverse transcription polymerase chain reaction (RT-PCR), flow cytometric analysis, and enzyme linked immunosorbent assay (ELISA). RT-PCR is used to measure cytokine at the transcriptional levels, while flow cytometric analysis are used to study intracellular cytokines and cell surface markers. Finally, ELISA is used to measure the amount of secreted cytokines in cell culture supernatants (Khalil et al., 2008).

Sachs et al. demonstrated that accumulation of eosinophils following IL-5 secretion, and to a lesser extent also with IL-10 and IFN-γ, from drug-specific stimulated T cells is a characteristic histological feature of drug-induced skin eruption. *In vitro* determination of drug-specific IL-5 secretion by peripheral blood mononuclear cells may be relevant for the detection of in drug-induced maculopapular exanthems (Sachs et al., 2002). CD69 is up-regulated after 48–72 h, and its determination by flow cytometry correlates with LTT for betalactames, sulphamethoxazole and carbamazepine HDR (Beeler et al., 2008). CD69 may be used for the evaluation of non-immediate reactions.

These *in vitro* tests represent important tools for diagnosis. They are, however, used mainly as research methods rather than as routine procedures. It must be also taken into consideration the timing of sample collection, which is critical as mediators can be secreted in transitory peaks with variants in the maintenance of detectable levels (Mayorga et al., 2006), and chemokines and cytokines can be degraded by protease (Niwa et al., 2000).

### Other *in vitro* tests

Other *in vitro* tests have been proposed for the study of drug allergy reactions. Among these the cellular allergen stimulation test (CAST-ELISA®) for the measurement of leukotrienes after peripheral blood leukocyte stimulation, basophil histamine release tests and a basophil activation test (BASO-Test®) can be mentioned. CAST-ELISA® is commercially available but it has not been sufficiently evaluated to recommend as a standard investigation outside the context of prospective studies. Basophil activation markers using fluorescence activated cell sorter analysis are currently being evaluated for certain type of drug allergic reactions but there seems to be no evidence currently of any advantage of these tests over skin testing (Mirakian et al., 2009).

## Potential *in vitro* pre-clinical tests to assess hypersensitivity

None of the *in vitro* methods mentioned above are, however, useful in preclinical safety assessment. It will important to have *in vitro* methods to screen drugs for their potential to induce hypersensitivity reactions. In the last decade an incredible progress has been made in the development of non-animal tests to assess contact hypersensitivity, and some tests have been formally validated. Methods based on the use of dendritic cells and the T cell priming are discussed below.

### The T cell priming assay

Chemicals can elicit T-cell-mediated diseases, including adverse drug reactions. The T cell priming assay (TCPA) was developed primarily for the identification of contact allergens within the integrated EU project SENS-IT-IV. This assay allows the detection of chemical-specific T cells in naive human peripheral T-cell population by measurement of proliferation and, at the single cell level, of IFN-γ production in CD4+ and CD8+ T cells. This assay may be a valuable, highly specific element in an integrated testing strategy for the predictive *in vitro* identification of contact allergens and possibly drugs that cause T cell-mediated adverse drug reactions (Dietz et al., 2010; Martin et al., 2010; Richter et al., 2013).

### Myeloid U937 skin sensitization test (MUSST) and modified MUSST (mMUSST)

The MUSST is an *in vitro* method proposed to assess skin sensitization. Dendritic cell activation following exposure to sensitizers was modeled in the U937 human myeloid cell line by measuring the induction of the expression of CD86 by flow cytometry after 48 h of chemical treatment. A test substance is predicted to have a dendritic cell activating potential indicative of being a sensitizer when CD86 induction exceeds the threshold of 1.5-fold with respect to vehicle treated cells at any tested concentration showing a cell viability ≥70 % in at least two independent experiments (Urbisch et al., 2015).

In the modified version of the MUSST (mMUSST), a test substance is predicted to have a dendritic cell activating potential when CD86 induction exceeds a threshold of 1.2-fold (Bauch et al., 2012). Among more than 145 substances available from these two *in vitro* assays, also some drug sensitizers, namely benzocaine, hydroquinone, p-benzoquinone and diphenylclopropenone were tested and resulted correctly classified with the MUSST (Bauch et al., 2012; Natsch et al., 2013; Urbisch et al., 2015).

### The human cell line activation test (h-CLAT)

The h-CLAT quantifies changes in CD86 and CD54 expression in the human THP-1 cell line following 24 h exposure to the test chemical. The changes in surface marker expression are measured by flow cytometry. Cytotoxicity measurement is conducted concurrently to assess whether up-regulation of surface maker expression occurs at sub-cytotoxic concentrations. The prediction model use the relative fluorescence intensity of surface markers compared to solvent control to discriminate between sensitizers and non-sensitizers. Among the 166 substances tested in this *in vitro* assay, the allergenic drugs benzocaine, clofibrate, pyridine, hydroquinone, p-benzoquinone and diphenylclopropenone resulted correctly classified (Nukada et al., 2012; Takenouchi et al., 2013; Urbisch et al., 2015).

Overall, even if the number of drugs tested is very limited, data suggests that pharmaceuticals may share with chemical allergens a common mechanism of action that activates dendritic cells, and support the possibility to use these *in vitro* methods also for the identification of drugs potentially associated with hypersensitivity reactions.

### The THP-1 activation assay

As mentioned above, most drugs are small molecules and are by themselves, not immunogenic. During the haptenization process, these small molecules bind to carrier proteins to form a complete immunogenic complex (Chang and Gershwin, 2010). The hypersensitivity reaction then requires the activation and maturation of dendritic cells (DCs), which will then drive the activation of specific T cells (Martin, 2012). DCs are antigen-presenting cells (APC) that play a central role in the initiation and regulation of adaptive immune responses. Following the contact with antigens, DCs undergo a process of maturation associated with the expression of several co-stimulatory molecules on the membrane such as CD80, CD86 and CD40, various adhesion molecules (CD2, CD11a, CD54, CD58), and secrete different cytokines, including IL-1β and IL-8 (Quah and O'Neill, 2005). Once activated, DCs migrate into the regional lymph node or in the spleen, where they present antigen to specific T lymphocytes, through MHC class II molecules (Ryan et al., 2007) and co-stimulatory adhesion molecules expressed on both DC (i.e., CD86) and T cell (i.e., CD28) to ensure the necessary contact to achieve full T-cell activation. Following stimulation, a clone of T cells is produce with the ability to react to the antigen, resulting in the clinical manifestation of HDR.

Within the European project SENS-IT-IV, we have previously established an *in vitro* method able to identify contact and respiratory allergens based on the use of the human THP-1 cell line (the same cell line used in the h-CLAT) and IL-8 release: the THP-1 activation assay (Mitjans et al., 2008, 2010). IL-8 is a potent chemotactic peptide for neutrophils as well as for T lymphocytes, basophils, and NK cells. In parallel to IL-8 production, several of the proposed *in vitro* methods, including the ones mentioned above, are based on DC and CD86 alone or in combination with CD54 expression for the identification of chemical sensitizers, due to their roles in antigen presentation and T cell activation.

**Figure 2:**
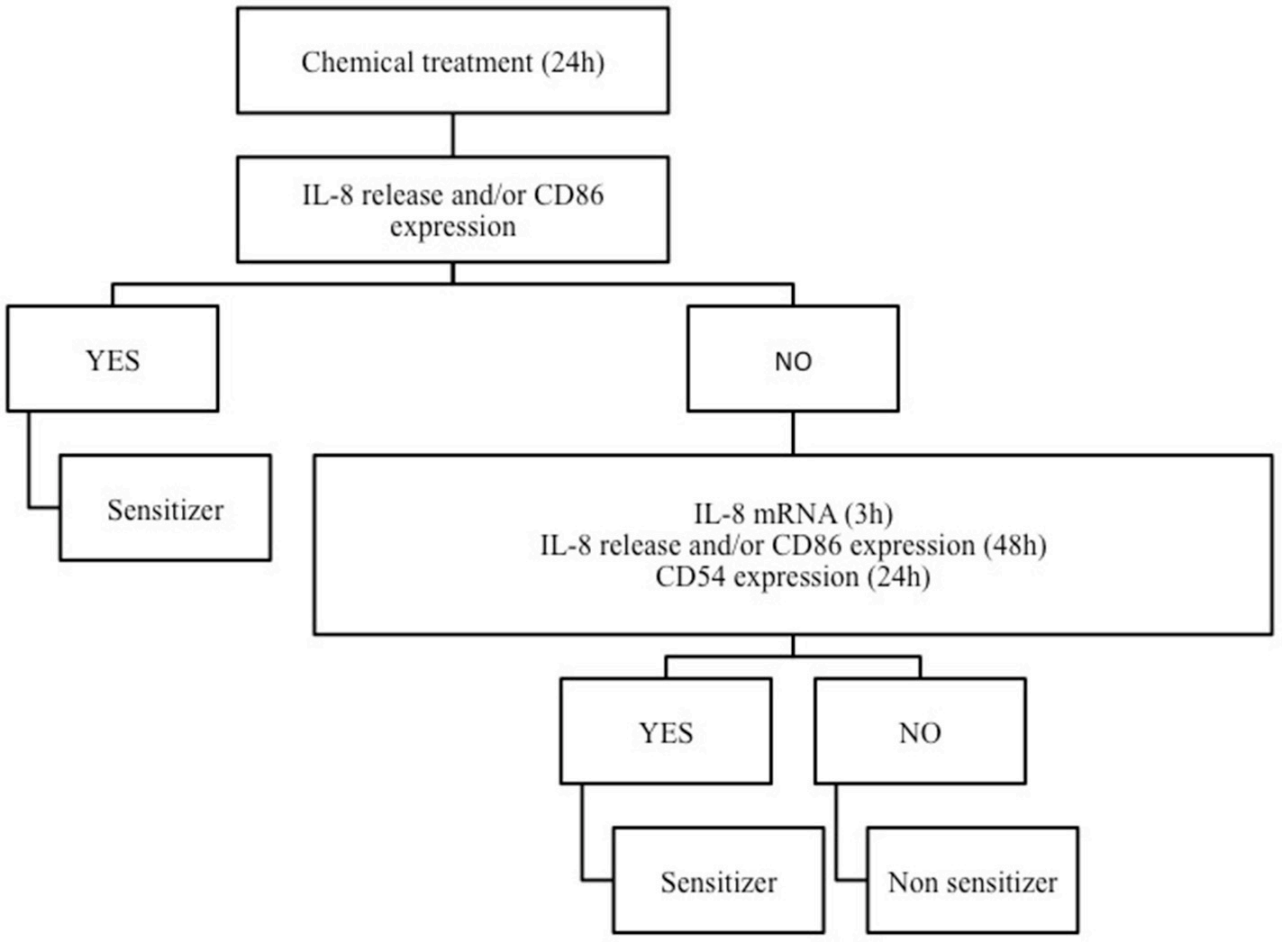
**The THP-1 activation assay tier approach**. Following 24 h of THP-1 chemical/drug treatment, the effect on IL-8 release and CD86 expression are investigated. If positive (statistically significant release of IL-8 at any concentration and/or a SI ≥ 1.5 for CD86), the chemical/drug will be considered as sensitizer. If negative, in order to exclude any activation, IL-8 release and CD86 expression at 48 h (statistically significant release of IL-8 at any concentration and/or a SI ≥ 1.5 for CD86) or CD54 expression at 24 h (SI ≥ 2.0) or alternatively IL-8 mRNA expression (2^−ΔΔCT^ > 3.0) at 3 h should be assessed. Only if negative results were obtained in all parameters, the chemical/drug will be considered as non-sensitizer.

Based on the notion that drug sensitizers and chemical sensitizers share the same mode of action, we recently investigated the possibility to use the THP-1 activation assay developed for skin and respiratory sensitizers, for the *in vitro* identification of pharmaceuticals, which may be associated with *in vivo* drug hypersensitivity reactions (Corti et al., 2015). It is well known that allergen drugs share with chemical allergens common mechanisms of cell activation and for reason we propose the THP-1 activation assay also for the hazard identification of immune-mediated hypersensitivity reactions induced by pharmaceuticals. Drugs were selected on the basis of clear *in vivo* immune-adverse reactions reported in literature, post-marketing data or labeling information, and on the commercial availability as pure drugs. Clonidine, ofloxacine, procainamide, streptozotocin, sulfamethoxazole which have been associated with a relatively high incidence of immune-mediated hypersensitivity reactions (Weaver et al., 2005), methyl salicylate and probenecid, which have been reported to cause irritant or allergic contact dermatitis and anaphylactic reactions (Corti et al., 2015), have been tested. We developed a strategy based on IL-8 production, CD86 and/or CD54 expression in THP-1 cells useful for the *in vitro* identification of drug sensitizers (see Figure 2). There are some important differences with the previous mentioned h-CLAT. First, the method we use to calculate CD86 and CD54 expression is different from h-CLAT protocol: in h-CLAT only the MFI is considered, while we also include the percentage of positive cells. The second point is the concentration tested. In fact, we observed that quite often a CV75 couldn't be reached with drugs compared to chemicals, meaning that drugs are less cytotoxic. The test we developed allowed the correct identification of all the selected drugs tested, including sulfamethoxazole, probenecid and procainamide for which metabolism is needed. Penicillin G, another drug frequently associated with hypersensitivity reactions (Siegel and Coleman, 1957), was previously tested, and found to be able to induced a dose-related release of IL-8 following 48 h of exposure (Mitjans et al., 2008).

Exposure of THP-1 cells to sensitizing drugs results in most cases in dose related release of IL-8 and increase in CD86 expression, with some differences among drugs, markers and times of exposure. As shown in Table 1, the combination of both IL-8 and CD86 expression allows the identification of all drugs tested. The use of IL-8 mRNA expression at 3 h or CD54 expression at 24 h may offer an alternative to the 48 h exposure and increase our confidence in the negativity of a drug (see Corti et al., 2015). The expression of IL-8 mRNA at 3 h is based on previous observation we made on chemical allergens failing to induce the release of IL-8: all chemicals sensitizers tested including pro-hapten induced IL-8 mRNA at 3 h (Galbiati et al., 2012).

**Table 1:** **Time of IL-8 release and CD86 expression of the selected drugs in THP-1 assay**.

**Chemical**	**CV_75_ (μg/ml)**	**Statistical significant IL-8 release**	**CD86 expression**
Streptozotocin	>2000	24 h	24 h
Sulfamethoxazole	>1000	48 h	48 h
Procainamide	>2000	24 h	24 h
Ofloxacin	>1000	24 h	24 h
Neomycin	>2000	24 h	–
Clonidine	750	24 h	24h
Methyl salicylate	>1000	24 h	24h
Probenecid	600	24 h	48h
Metformin	>2000	–	–

10^6^/ml cells were treated for 24–48 h with increasing concentrations of the selected drugs. Cell viability was assessed by PI staining. CV75 (the concentration resulting in 75% of cells viability compared to vehicle treated cells) was calculated by linear regression analysis of data. IL-8 release was measured by ELISA. CD86 expression was evaluated by flow cytometric analysis. Original data are present in Corti et al. (2015).

Legend: – no induction observed.

Using streptozotocin as reference drug to study the mechanisms of action, we could demonstrate a key role for p38 mitogen-activated protein kinase (p38 MAPK) and PKC-β activation in streptozotocin-induced IL-8 release and CD86 expression (Corti et al., 2015), confirming previous results obtained with chemical allergens (Mitjans et al., 2008; Corsini et al., 2014).

**Figure 3:**
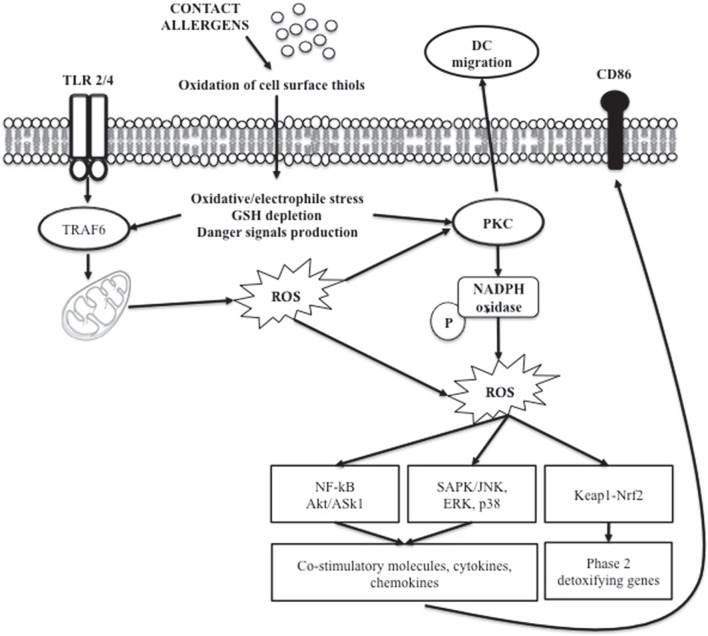
**Role of ROS in chemical allergen-induced DC activation**.

Evidence indicates that oxidative stress is involved in chemical-induced skin allergic and inflammatory diseases (Okayama, 2005; Byamba et al., 2010). Chemical-induced oxidation of the cell surface thiols appears to be one of the triggers of DC maturation, resulting in intracellular redox imbalance that generate stress-related signal (Figure 3). The Keap1/Nrf2-signaling pathway is dedicated to the detection of electrophilic stress in cells leading to the up-regulation of genes involved in protection or neutralization of chemicals reactive species (Natsch and Emter, 2007). It has been shown in human monocyte-derived dendritic cells that chemical sensitizers induced oxidative stress measured by the glutathione GSH/GSSG ratio, as a redox marker (Mizuashi et al., 2005). The reduction of the glutathione GSH/GSSG ratio was accompanied by CD86 up-regulation and p38 MAPK activation, suggesting that the electrophilic properties of chemicals sensitizers may be perceived by DCs as a danger signal leading to DC maturation (Sasaki and Aiba, 2007). Engagement of certain Toll like receptors (TLR1, 2, and 4) leads to mitochondrial translocation of the signal adaptor TRAF6. At the mitochondria, TRAF6 interacts with ECSIT, a protein implicated in the assembly of complex I, leading to its ubiquitylation, which results in increased ROS production. Proteins that are reversibly modulated by ROS are of high interest. In this context, protein kinases and phosphatases, which act co-ordinately in the regulation of signal transduction through the phosphorylation and dephosphorylation of target proteins, have been described to be key elements in ROS-mediated signaling events. In particular, PKC isoforms have been shown to contain a unique structural feature that is susceptible to oxidative modification (Cosentino-Gomes et al., 2012). The high levels of cysteine residues render the regulatory domain susceptible to redox regulation (Gopalakrishna and Jaken, 2000; Giorgi et al., 2010). Currently, evidence supports the direct activation of different PKC isoforms by ROS generation; in particular the β isoform is able to induce ROS generation through mitochondrial damage (Pinton et al., 2007).

To summarize the drugs tested in the preclinical available *in vitro* methods and known to be able to induce hypersensitivity reactions, a list is reported in Table 2.

**Table 2:** **Drugs known to induce hypersensitivity and resulted positive in DC-based *in vitro* methods**.

**Drug**	**h-CLAT**	**MUSST**	**mMUSST**	**THP-1 activation assay**
Benzocaine	^*^	#		
Clofibrate	x			
Pyridine	^*^			
Hydroquinone	^*^	#		
p-Benzoquinone	^*^	#	+	
Diphenylclopropenone	^*^	#		
Streptozotocin				§
Sulfamethoxazole				§
Procainamide				§
Ofloxacin				§
Neomycin				§
Clonidine				§
Methyl salicylate				§
Probenecid				§
Metformin				§

^*^, Nukada et al. (2012); x, Takenouchi et al. (2013); #, Natsch et al. (2013); +, Bauch et al. (2012); §, Corti et al. (2015).

## Conclusion

Develop a new pharmaceutical entity has many possibilities of failure and is a very expensive process. Methods and/or models to assess the hazard of hypersensitivity reactions by pharmaceuticals are not yet validated (or requested on a routine basis). The assessment of hypersensitivity of pharmaceuticals would benefit from a rationalistic approach using clear and linear test methods that are based on immunological knowledge and read out parameters. There are many *in vitro* tests that can help in diagnosis and identification of the pharmaceuticals able to cause a hypersensitivity reaction, and among of these, we established, using the THP-1 cell line as surrogate of dendritic cells, experimental conditions and markers to correctly identify drug sensitizers. The THP-1 cells assay can be easily incorporated into drug development to identify drugs that may have the potential to cause systemic hypersensitivity reactions.

## Author contributions

VG wrote the manuscript; AP, and EK performed the experiments depicted in Figure 3; EC supervised the overall project and finalized the final version of the review. All authors have read this review and gave their agreement for submission.

### Conflict of interest statement

The authors declare that the research was conducted in the absence of any commercial or financial relationships that could be construed as a potential conflict of interest. The reviewer NV and handling Editor declared their shared affiliation, and the handling Editor states that the process nevertheless met the standards of a fair and objective review.

## References

[B1] Albers R. Broeders A. van der Pijl A. Seinen W. Pieters R. (1997). The use of reporter antigens in the popliteal lymph node assay to assess immunomodulation by chemicals. Toxicol. Appl. Pharmacol. 143, 102–109. 10.1006/taap.1996.8078 9073598

[B2] Bauch C. Kolle S. N. Ramirez T. Eltze T. Fabian E. Mehling A.. (2012). Putting the parts together: combining *in vitro* methods to test for skin sensitizing potentials. Regul. Toxicol. Pharmacol. 63, 489–504. 10.1016/j.yrtph.2012.05.013 22659254

[B3] Beeler A. Pichler W. J. (2007). *In vitro* tests of T-cell mediated drug hypersensitivity, in Drug Hypersensitivity, ed Pichler W. J. (Basel: Karger), 380–390.

[B4] Beeler A. Zaccaria L. Kawabata T. T. Gerber B. O. Pichler W. J. (2008). CD69 upregulation on T cells as an *in vitro* marker for delayed-type drug hypersensitivity. Allergy 62, 181–188. 10.1111/j.1398-9995.2007.01516.x 18005225

[B5] Byamba D. Kim T. G. Kim D. H. Je J. H. Lee M. G. (2010). The roles of reactive oxygen species produced by contact allergens and irritants in monocyte-derived dendritic cells. Ann. Dermatol. 22, 269–278. 10.5021/ad.2010.22.3.269 20711262 PMC2917679

[B6] Chang C. Gershwin M. E. (2010). Drugs and autoimmunity–a contemporary review and mechanistic approach. J. Autoimmun. 34, J266–J275. 10.1016/j.jaut.2009.11.012 20015613

[B7] Corsini E. Galbiati V. Esser P. R. Pinto A. Racchi M. Marinovich M.. (2014). role of PKC-β in chemical allergen-induced CD86 expression and IL-8 release in THP-1 cells. Arch. Toxicol. 88, 415–424. 10.1007/s00204-013-1144-z 24136171

[B8] Corti D. Galbiati V. Gatti N. Marinovich M. Galli C. L. Corsini E. (2015). Optimization of the THP-1 activation assay to detect pharmaceuticals with potential to cause immune mediated drug reactions. Toxicol. In Vitro 29, 1339–1349. 10.1016/j.tiv.2015.04.012 26028146

[B9] Cosentino-Gomes D. Rocco-Machado N. Meyer-Fernandes J. R. (2012). Cell signaling through protein kinase C oxidation and activation. Int. J. Mol. Sci. 13, 10697–10721. 10.3390/ijms130910697 23109817 PMC3472709

[B10] Dietz L. Esser P. R. Schmincker S. S. Goette I. Richter A. Schno Izer M. (2010). Tracking human contact allergens: from mass spectrometric identification of peptide-bound reactivity small chemicals to chemical-specific naïve human T-cell priming. Toxicol. Sci. 117, 336–347. 10.1093/toxsci/kfq209 20631061

[B11] Galbiati V. Carne A. Mitjans M. Galli C. L. Marinovich M. Corsini E. (2012). Isoeugenol destabilizes IL-8 mRNA expression in THP-1 cells through induction of the negative regulator of mRNA stability tristetraprolin. Arch. Toxicol. 86, 239–248. 10.1007/s00204-011-0758-2 21969073

[B12] Giorgi C. Agnoletto C. Baldini C. Bononi A. Bonora M. Marchi S.. (2010). Redox control of protein kinase C: cell and disease-specific aspects. Antioxid. Redox Signal. 13, 1051–1085. 10.1089/ars.2009.2825 20136499

[B13] Gopalakrishna R. Jaken S. (2000). Protein kinase C signaling and oxidative stress. Free Radical. Biol. Med. 9, 1349–1361. 10.1016/S0891-5849(00)00221-5 10924854

[B14] Gruchalla R. S. (2001). Drug metabolism, danger signals, and drug-induced hypersensitivity. J. Allergy Clin. Immunol. 108, 475–488. 10.1067/mai.2001.118509 11590368

[B15] Hammond E. Almeida C. A. Mamotte C. Nolan D. Philipps E. Schollaardt T. A.. (2007). External quality assessment of HLA-B^*^5701 reporting: an international multicentre survey. Antivir. Ther. 12, 1027–1032. 18018760

[B16] Khalil G. El-Sabban M. Al-Ghadban S. Azzi S. Shamra S. Khalifé S.. (2008). Cytokine expression profile of sensitized human T lymphocytes following *in vitro* stimulation with amoxicillin. Eur. Cytokine Netw. 19, 131–141. 10.1684/ecn.2008.0132 18775806

[B17] Khan D. A. Solensky R. (2010). Drug allergy. J. Allergy Clin. Immunol. 125(Suppl. 2), S126–S137. 10.1016/j.jaci.2009.10.028 20176256

[B18] Kubota Y. Imayama S. Toshitani A. Miyahara H. Tahahashi T. Uemura Y.. (1997). Sulfidoleukotriene realease test (CAST) in hypersensitivity to nonsteroidal anti-inflammatory drugs. Int. Arch. Allergy Immunol. 114, 361–366. 10.1159/000237695 9414140

[B19] Lanzavecchia A. Sallusto F. (2000). Dynamics of T lymphocyte responses: intermediates, effectors, and memory cells. Science 290, 92–97. 10.1126/science.290.5489.92 11021806

[B20] Mallal S. Phillips E. Carosi G. Molina J. M. Workman C. Tomazic J.. (2008). HLA-B^*^5701 screening for hypersensitivity to abacavir. N. Engl. J. Med. 358, 568–579. 10.1056/NEJMoa0706135 18256392

[B21] Martin A. M. Frueger R. Almeida C. A. Nolan D. Philipps E. Mallal S. (2006). A sensitive and rapid alternative to HLA typing as a genetic screening test for abacavir hypersensitivity syndrome. Pharmacogenet. Genomics 16, 353–357. 10.1097/01.fpc.0000197468.16126.cd 16609367

[B22] Martin S. F. (2012). Allergic contact dermatitis: xenoinflammation of the skin. Curr. Opin. Immunol. 24, 720–729. 10.1016/j.coi.2012.08.003 22980498

[B23] Martin S. F. Esser P. R. Schmincker S. Dietz L. Naisbitt D. J. Park B. K. (2010). T-cell recognition of chemicals, protein allergens and drugs: towards the development of *in vitro* assay. Cell. Mol. Life Sci. 67, 4171–4184. 10.1007/s00018-010-0495-3 20717835 PMC11115584

[B24] Mayorga C. Celik G. Rouzaire P. Whitaker P. Bonadonna P. Cernadas J. R.. (2016). *In vitro* tests for drug hypersensitivity reactions. An ENDA/EAACI drug allergy interest group position paper. Allergy. 10.1111/all.12886. [Epub ahead of print]. 26991315

[B25] Mayorga C. Pena R. R. Blanca-López N. López S. Martin E. Torres M. J. (2006). Monitoring the acute ohase response in non-immediate allergic drug reactions. Curr. Opin. Allergy Clin. Immunol. 6, 249–257. 10.1097/01.all.0000235897.72429.4a 16825864

[B26] Mirakian R. Ewan P. W. Durham S. R. Youlten L. J. Dugué P. Friedmann P. S.. (2009). BSACI guidelines for the management of drug allergy. Clin. Exp. Allergy. 39, 43–61. 10.1111/j.1365-2222.2008.03155.x 19128352

[B27] Mitjans M. Galbiati V. Lucchi L. Viviani B. Marinovich M. Galli C. L.. (2010). Use of IL-8 release and p38 MAPK activation in THP-1 cells to identify allergens and to assess their potency *in vitro*. Toxicol. In Vitro 24, 1803–1809. 10.1016/j.tiv.2010.06.001 20541004

[B28] Mitjans M. Viviani B. Lucchi L. Galli C. L. Marinovich M. Corsini E. (2008). Role of p38 MAPK in the selective release of IL-8 induced by chemical allergen in naive THp-1 cells. Toxicol. In Vitro 22, 386–395. 10.1016/j.tiv.2007.10.005 18494145

[B29] Mizuashi M. Ohtani T. Nakagawa S. Aiba S. (2005). Redox imbalance induced by contact sensitizers triggers the maturation of dendritic cells. J. Invest. Dermatol. 124, 579–586. 10.1111/j.0022-202X.2005.23624.x 15737199

[B30] Natsch A. Emter R. (2007). Skin sensitizers induce antioxidant response element dependent genes: application to the *in vitro* testing of the sensitization potential of chemicals. Toxicol. Sci. 102, 110–119. 10.1093/toxsci/kfm259 17932397

[B31] Natsch A. Ryan C. A. Foertsch L. Emter R. Jaworska J. Gerberick F.. (2013). A dataset on 145 chemicals tested in alternative assays for skin sensitization undergoing prevalidation. J. Appl. Toxicol. 33, 1337–1352. 10.1002/jat.2868 23576290

[B32] Niwa Y. Akamatsu H. Sumi H. Ozaki Y. Abe A. (2000). Evidence of degradation of cytokines in the serum of patients with atopic dermatitis by calcium-dependent protease. Arch. Dermatol. Res. 292, 391–396. 10.1007/s004030000148 10994773

[B33] Nukada Y. Ashikaga T. Miyazawa M. Hirota M. Sakaguchi H. Sasa H.. (2012). Prediction of skin sensitization potency of chemicals by human cell line activation test (h-CLAT) and an attempt at classifying skin sensitization potency. Toxicol. In Vitro 26, 1150–1160. 10.1016/j.tiv.2012.07.001 22796097

[B34] Nyfeler B. Pichler W. J. (1997). The lymphocyte transformation test for the diagnosis of drug allergy: sensitivity and specific. Clin. Exp. Allergy 27, 175–181. 10.1111/j.1365-2222.1997.tb00690.x 9061217

[B35] Okayama Y. (2005). Oxidative stress in allergic and inflammatory skin diseases. Curr. Drug Targets Inflamm. Allergy 4, 517–519. 10.2174/1568010054526386 16127829

[B36] Pâris-Köhler A. Demoly P. Persi L. Lbel B. Bousquet J. Arnoux B. (2000). *In vitro* diagnosis of cypress allergy by using cytofluorimetric analysis of basophils (Basotest). J. Allergy Clin. Immunol. 105, 339–345. 10.1016/S0091-6749(00)90085-X 10669856

[B37] Philipps E. J. (2006). The pharmacogenetics of antiretroviral therapy. Curr. Opin. HIV AIDS 1, 249–256. 10.1097/01.COH.0000221600.64659.d3 19372817

[B38] Pichler W. J. (2002). Pharmacological interaction of drugs with antigen-specific immune receptors: the p-i concept. Curr. Opin. Allergy Clin. Immunol. 2, 301–305. 10.1097/00130832-200208000-00003 12130944

[B39] Pichler W. J. (2003a). Delayed drug hypersensitivity reactions. Ann. Intern. Med. 139, 683–693. 14568857 10.7326/0003-4819-139-8-200310210-00012

[B40] Pichler W. J. (2003b). Drug-induced autoimmunity. Curr. Opin. Allergy Clin. Immunol. 3, 249–253. 10.1097/01.all.0000083955.99396.25 12865767

[B41] Pieters R. (2001). The popliteal lymph node assay: a tool for predicting drug allergies. Toxicology 158, 65–69. 10.1016/S0300-483X(00)00409-1 11164994

[B42] Pieters R. (2007). Detection of autoimmunity by pharmaceuticals. Methods 41, 112–117. 10.1016/j.ymeth.2006.09.005 17161307

[B43] Pinton P. Rimessi A. Marchi S. Orsini F. Migliaccio E. Giorgio M.. (2007). Protein kinase C beta and prolyl isomerase 1 regulate mitochondrial effects of the life-span determinant p66Shc. Science 315, 659–663. 10.1126/science.1135380 17272725

[B44] Porebski G. Pecaric-Petkovic T. Groux-Keller Bosek, M. Kawabata T. T. (2013). *In vitro* drug causality assessment in Stevenson-Johnson syndrome–alternatives for lymphocyte transformation test. Clin. Exp. Allergy 43, 1027–1037. 10.1111/cea.12145 23957338

[B45] Quah B. J. C. O'Neill H. C. (2005). Maturation of function in dendritic cells for tolerance and immunity. J. Cell. Mol. Med. 9, 643–654. 10.1111/j.1582-4934.2005.tb00494.x 16202211 PMC6741309

[B46] Ravel G. Descotes J. (2005). Popliteal lymph node assay: facts and perspectives. J. Appl. Toxicol. 25, 451–458. 10.1002/jat.1072 15986413

[B47] Richter A. Schmincker S. S. Esser P. R. Traska V. Weber V. Dietz L.. (2013). Human T cell priming assay (hTCPA) for the identification of contact allergens based on naïve T cell and DC-IFN-γ and TNF-α readout. Toxicol. In Vitro 27, 1180–1185. 10.1016/j.tiv.2012.08.007 22906571

[B48] Riedl M. A. Casillas A. M. (2003). Adverse drug reactions: types and treatment options. Am. Fam. Physician 68, 1781–1790. 14620598

[B49] Romano A. Blanca M. Torres M. J. Bircher A. Abere W. Brokow K.. (2004). Diagnosis of nonimmediate reactions to beta-lactam antibiotics. Allergy 59, 1553–1560. 10.1111/j.1398-9995.2004.00678.x 15461594

[B50] Romano A. Torres M. J. Quaratino D. Di Fonso M. Perrone M. R. Viola M. (1997). Diagnostic evaluation of delayed hypersensitivity to systematically administered drugs. Allergy 27, 175–181. 10735645

[B51] Ryan C. A. Kimber I. Basketter D. A. Pallardy M. Gildea L. A. Gerberick G. F. (2007). Dendritic cells and skin sensitization: biological role and uses in hazard identification. Toxicol. Appl. Pharmacol. 221, 37896–37903. 10.1016/j.taap.2007.03.006 17493650

[B52] Sachs B. Al Massaoudi T. Merk H. F. Erdmann S. (2004). Clinical and laboratory investigations. Combined *in vivo* and *in vitro* approach for the characterization of penicillin-specific polyclonal lymphocyte reactivity: tolerance tests with safe penicillins instead of challenge with culprit drugs. Br. J. Dermatol. 151, 809–816. 10.1111/j.1365-2133.2004.06238.x 15491421

[B53] Sachs B. Erdmann S. Malte Baron J. Neis M. Al Masaoudi T. Merk H. F. (2002). Determination of interleukin-5 secretion from drug-specific activated *ex vivo* peripheral blood mononuclear cells as a test system for the *in vitro* detection of drug sensitization. Clin. Exp. Allergy 32, 736–744. 10.1046/j.1365-2222.2002.01382.x 11994099

[B54] Sasaki Y. Aiba S. (2007). Dendritic cells and contact dermatitis. Clin. Rev. Allergy Immunol. 33, 27–34. 10.1007/s12016-007-0034-7 18094944

[B55] Siegel B. B. Coleman M. (1957). Studies in penicillin hypersensitivity. IV. Antigenic properties of altered procaine penicillin. J. Allergy 28, 264–271. 10.1016/0021-8707(57)90132-6 13428490

[B56] Sullivan A. Gibson A. Park B. K. Naisbitt D. J. (2015). Are drug metabolites able to cause T-cell mediated hypersensitivity reactions? Expert Opin. Drug Metab. Toxicol. 11, 357–368. 10.1517/17425255.2015.992780 25495340

[B57] Takenouchi O. Miyazawa M. Saito K. Ashikaga T. Sakaguchi H. (2013). Predictive performance of the human Cell Line Activation Test (h-CLAT) for lipophilic chemicals with high octanol-water partition coefficients. J. Toxicol. Sci. 38, 599–609. 10.2131/jts.38.599 23824015

[B58] Torres M. J. Blanca M. Fernandez J. Romano A. Weck A. Abere W.. (2003). Diagnosis of immediate allergic reactions to beta-lactam antibiotics. Allergy 58, 961–972. 10.1034/j.1398-9995.2003.00280.x 14510712

[B59] Urbisch D. Mehling A. Guth K. Ramirez T. Honarvar N. Kolle S.. (2015). Assessing skin sensitization hazard in mice and men using non-animal test methods. Regul. Toxicol. Pharmacol. 71, 337–351. 10.1016/j.yrtph.2014.12.008 25541156

[B60] Warbrick E. V. Dearman R. J. Kimer I. (2001). Prediction of drug allergenicity: possible use of the local lymph node assay. Curr. Opin. Drug Discov. Devel. 4, 60–65. 11727324

[B61] Warrington R. Silviu-Dan F. (2011). Drug allergy. Allergy Asthma Clin. Immunol. 7, S10. 10.1186/1710-1492-7-S1-S10 22165859 PMC3245433

[B62] Weaver J. L. Chepdelaine J. M. Descotes J. Germolec D. Holsapple M. House R.. (2005). Evaluation of a lymph node proliferation assay for its ability to detect pharamceuticals with potential to cause immune-mediated drug reactions. J. Immunotoxicol. 2, 11–20. 10.1080/15476910590930100 18958655

[B63] Weltzien H. U. Moulon S. Martin S. Padovan E. Hartmann U. Kohler K. (1996). T cell immune responses to haptens. Structural models for allergic and autoimmune reactions. Toxicology 107, 141–151. 10.1016/0300-483X(95)03253-C 8599173

[B64] Zawodniak A. Lochmatter P. Yerly D. Kawabata T. T. Lerch M. Yawalker N. (2010). *In vitro* detection of cytotoxic T and NK cells in peripheral blood of patient with various drug-induced skin diseases. Allergy 65, 376–384. 10.1111/j.1398-9995.2009.02180.x 19793058

